# Temozolomide with Radiation Therapy in High Grade Brain Gliomas: Pharmaceuticals Considerations and Efficacy; A Review Article

**DOI:** 10.3390/molecules14041561

**Published:** 2009-04-16

**Authors:** Georgios V. Koukourakis, Vassilios Kouloulias, Georgios Zacharias, Christos Papadimitriou, Panagiotis Pantelakos, George Maravelis, Andreas Fotineas, Ivelina Beli, Demetrios Chaldeopoulos, John Kouvaris

**Affiliations:** 1 Attikon University Hospital of Athens, Second Radiology Department, Radiation Therapy Unit, Medical School of Athens, Greece; E-mails: vkouloul@cc.ece.ntua.gr (V.K.), georgios_zaharias@yahoo.gr (G.Z.), panpan@otenet.gr (P.P.), maravelis@gmail.com (G.M.), viridan55@hotmail.com (A.F.), kbelis@in.gr (I.B.), dhal@her.forthnet.gr (D.C.); 2 University of Athens, Medical Oncology Unit, Alexandra Hospital Athens Greece; E-mail: chrpapadim@yahoo.gr (C.P.); 3 Aretaieion University Hospital, 1st Radiology Department, Radiation Therapy Unit, Medical School of Athens, Greece; E-mail: johnkouv@aretaieio.uoa.gr (J.K.)

**Keywords:** Temozolomide, Malignant gliomas, Chemotherapy, Radiation therapy

## Abstract

Malignant gliomas (glioblastoma multiforme and anaplastic astrocytoma) which have a combined incidence of 5–8/100,000 population, represent the most common primary central nervous system tumors. The treatment outcomes even with aggressive approach including surgery, radiaton therapy and chemotherapy are dismal with median reported survival is less than 1 year. Temozolomide is a new drug which has shown promise in treating malignant gliomas and other difficult-to-treat tumors. This drug is a per os (p.o) imidazotetrazine second-generation alkylating agent which represents the leading compound in a new class of chemotherapeutic agents that enter the cerebrospinal fluid and do not require hepatic metabolism for activation.

The efficacy of temozolomide was tested *in vitro* studies and has demonstrated schedule-dependent antitumor activity against highly resistant malignancies, including high-grade glioma (HGG). In addition, in clinical studies, temozolomide consistently demonstrates reproducible linear pharmacokinetics with approximately 100% p.o. bioavailability, noncumulative minimal myelosuppression that is rapidly reversible, and activity against a variety of solid tumors in both children and adults. Moreover, preclinical studies have evaluated the combination of temozolomide with other alkylating agents and inhibitors of the DNA repair protein *O*^6^-alkylguanine alkyltransferase to overcome resistance to chemotherapy in malignant glioma and malignant metastatic melanoma. At the present time temozolomide is approved in the United States for the treatment of adult patients with refractory anaplastic astrocytoma and, in the European Union, for treatment of glioblastoma multiforme showing progression or recurrence after standard therapy. Temozolomide’s characteristics which make it a candidate for a wide range of clinical testing to evaluate the potential of combination treatments in different tumor types are its predictable bioavailability and minimal toxicity. An overview of the mechanism of action of temozolomide and a summary of results from more important randomized controlled clinical trials in high grade gliomas are presented here.

## Introduction

Temozolomide (TMZ; Temodar™ [Temodal™ in the United Kingdom and Europe], Schering-Plough Corporation; Kenilworth, NJ) is a novel oral alkylating agent that has shown efficacy in the treatment of a variety of solid tumors, including primary malignant brain tumors [1,2,3,4,5,6,7]. TMZ has certain advantages over many existing alkylating agents because of its unique chemical structure and pharmacokinetic properties [8,9]. Because of its small molecular weight, TMZ efficiently crosses the blood brain barrier and is effective against primary brain tumors [10]. In addition, TMZ can be administered orally without dietary restrictions, and essentially 100% of the orally administered dose enters the blood flow. TMZ is also associated with a low incidence of severe adverse events. Unlike nitrosoureas and other alkylating agents that chemically cross-link the DNA and are associated with severe, doselimiting, cumulative hematologic toxicity, TMZ is associated with generally mild, noncumulative myelosuppression.

In Phase 1 and 2 clinical studies conducted by the CRC (London, United Kingdom), temozolomide was absorbed rapidly, exhibited 100% p.o. bioavailability within 1–2 h of administration, and demonstrated antineoplastic activity in recurrent high-grade glioma [11,12,13,14]. Results of these trials showed that when temozolomide is administered p.o. once daily for 5 days in a 4-week cycle, it is well tolerated, producing mild-to-moderate toxicity that is both expected and easily managed. The results also confirmed the ability of temozolomide to penetrate the CNS (central nervous system) and indicated that temozolomide has considerable potential in treating gliomas and improving the QOL (quality of life) of patients with glioma [13,14,15]. Additional Phase 1 studies have confirmed these results and have extended these observations to pediatric patients [16,17].

Temozolomide has been evaluated in a number of Phase 2 and 3 clinical trials for the treatment of glioblastoma multiforme and anaplastic astrocytoma, malignancies for which there are no satisfactory therapies. On the basis of the results of these studies, temozolomide has been approved in the European Union for the treatment of patients with glioblastoma multiforme showing progression or recurrence after standard therapy. Moreover, temozolomide received accelerated approval from the FDA (food and drug administration) for treatment of adult patients with anaplastic astrocytoma who have relapsed after treatment that included a nitrosurea drug (BCNU or CCNU) and procarbazine. Recently, FDA gave full approval for temozolomide as first line treatment for glioblastoma, a decision followed by the EMEA (European Medicines Agency). Studies are under way to evaluate the combination of temozolomide with other chemotherapeutic agents and biochemotherapy in the treatment of malignant glioma, respectively. This article reviews the mechanism of action of temozolomide as an anticancer agent and summarizes the most recent clinical studies of temozolomide for the treatment of malignant gliomas.

## Materials and Methods

### Identification of Eligible Studies

We searched MEDLINE and the Cochrane Central Register of Controlled Trials (last search on January 2009) using combinations of terms, such as: temozolomide, malignant gliomas, radiation therapy and treatment. We considered all meta-analysis or randomized controlled trials providing information about the effectiveness of temozolomide on treatment of malignant gliomas, its adverse profile effects, its dosage and administration, and future directions of ongoing research as eligible.

### Data Extraction

We have incorporated as eligible in this review all randomized controlled trails that had two arms: first arm, intervention, patients that had received radiation therapy comcomitant with temozolomide and second arm, comparison, patients that had received radiation therapy only or radiation therapy in association with other form of chemotherapy e.g PCV, Gliadel. The primary outcomes were survival from time to randomization to time of death, and secondary were: time to progression, quality of life and adverse events. We have extracted information from each eligible study. The data recorded, included author’s name, year of publication, number of patients included in the study, treatment schedule, percentage overall response, median time to progression and median survival.

## Chemical structure and mechanism of action

**Figure 1:**
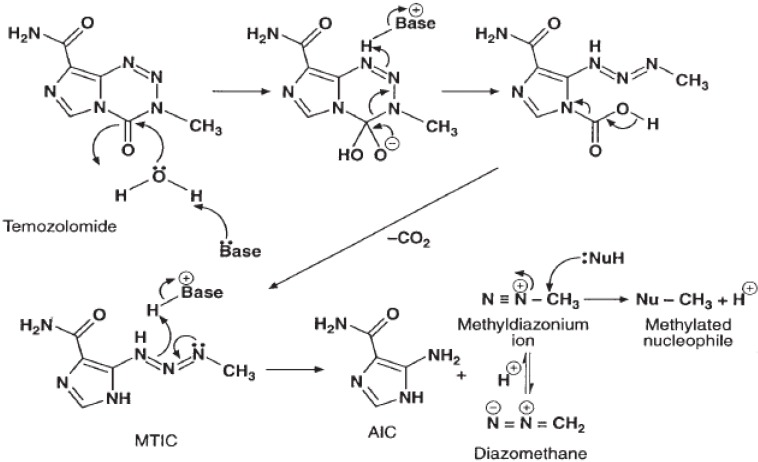
Metabolism of temozolomide to its active form.

TMZ belongs to the imidazotetrazine family, which is a new class of alkylating agents. These compounds contain an imidazole ring and are structurally and functionally similar to DTIC (dacarbazine), which is a member of the triazene subgroup [9]. TMZ was first synthesized by Stevens and coworkers in 1984 [8], and they were the first to demonstrate that TMZ had anticancer activity [8,18]. TMZ is a small molecule with a molecular weight of 194 Daltons and is, therefore, readily absorbed in the digestive tract and, because it is lipophilic, it is able to cross the blood-brain barrier. TMZ is highly stable at the acidic pH of the stomach. Nevertheless, once in contact with the slightly basic pH of the blood and tissues, TMZ spontaneously undergoes hydrolysis to the active metabolite MTIC [5-(3-dimethyl-1-triazenyl)imidazole-4-carboxamide], which rapidly breaks down to form the reactive methyldiazonium ion (Figure 1) [9]. Penetration of TMZ into the CNS has been studied in rats and rhesus monkeys, and these studies have shown that the levels of drug in the brain and cerebrospinal fluid are approximately 30% to 40% of the plasma concentration [10]. The first human pharmacokinetic study on TMZ to quantify CSF penetration was conducted by Ostermann *et al.* [19]. They have assessed TMZ pharmacokinetics in plasma and cerebrospinal fluid (CSF) along with its interindividual variability, to characterize covariates and to explore relationships between systemic or cerebral drug exposure and clinical outcomes. The authors have concluded that the area under the concentration-time curve (AUC), AUC_CSF_/AUC_plasma_ ratio was 20% and that systemic or cerebral exposures are not better predictors than the cumulative dose alone for both efficacy and safety. The metabolite MTIC, however, does not effectively penetrate the CNS.

The methyldiazonium ion formed by the breakdown of MTIC primarily methylates guanine residues in the DNA molecule, resulting in the formation of *O*^6^- and *N*^7^-methylguanine. The formation of *O*6-methylguanine is primarily responsible for the cytotoxic effects of TMZ and DTIC. When DNA mismatch repair enzymes attempt to excise *O*^6^-methylguanine, they generate single- and double-strand breaks in the DNA, leading to activation of apoptotic pathways. *N*^7^-methylguanine is comparatively less cytotoxic because the nucleotide excision repair pathway can successfully excise these adducts without damaging the DNA. TMZ does not result in chemical cross-linking of the DNA strands. Thus, it is less toxic to the hematopoietic progenitor cells in the bone marrow than are the nitrosoureas (i.e., carmustine, lomustine), platinum compounds, and procarbazine, which do cross-link the DNA.

## Mechanisms of Resistance

The two primary mechanisms of resistance for temozolomide and other alkylating agents are the enzyme AGT (alkylguanine alkyltransferase) [20,21] and a deficiency in the MMR (mismatch repair ) pathway. Of these two mechanisms, AGT plays a primary role in resistance to temozolomide by removing the alkyl groups from the *O*^6^ position of guanine, in effect reversing the cytotoxic lesion of temozolomide [22]. The sensitivity of tumor cell lines to temozolomide and the alkylating agents BCNU and DTIC can be correlated with AGT levels [24,25,26,27,28]. Moreover, retrovirus-mediated transfer of human AGT gene to cells that are devoid of endogenous AGT activity confers a high level of resistance on temozolomide and other methylating and chloroethylating agents.

Despite the fact that AGT is clearly important in the resistance of cells to temozolomide, some cell lines that express low levels of AGT are nevertheless resistant, which suggests that other mechanisms for resistance may be implicated [29,30]. A insufficiency in the MMR pathway succeeded from mutations in any one or more of the five or six protein complexes that identify and correct DNA can ascribe cells tolerant to methylation and to the cytotoxic effects of temozolomide. This deficiency in the MMR pathway results in a failure to recognize and repair the *O*^6^-MG adducts produced by temozolomide and other methylating agents [31,32,33]. DNA replication continues past the *O*^6^-MG adducts without cell cycle arrest or apoptosis. Resistance in tumor cells that are deficient in MMR is unrelated to the level of AGT and is, therefore, unaffected by AGT inhibitors.

Another possible mechanism of resistance for temozolomide is the base excision repair pathway. Studies have shown that treatment of human tumor cells with temozolomide induced an increase in the activity of PARP (poly(ADP)-ribose polymerase), which is believed to be involved in nucleotide excision repair [34,35], and the inhibition of PARP has been reported to enhance the cytotoxicity of methylating agents [36,37,38]. Several studies with inhibitors of PARP and with cell lines deficient in either MMR or excision repair have indicated a role of the repair of *N*^7^-methylguanine and *O*^3^-methyladenine adducts in the resistance to the antitumor activity of temozolomide and other alkylating agents [33,36,37,39]. However, the importance of these adducts in the antitumor activity of the drug may be secondary to that of the *O*^6^-MG adduct, except in those tumors that are deficient in base excision repair [40,41,42].

## Clinical pharmacology

TMZ is rapidly absorbed after oral administration, and the bioavailability is approximately 100%. The absorption of TMZ is only minimally affected by food. Absorption is reduced by only approximately 9% when taken with food, and this is not a clinically significant effect [43]. However, to reduce potential for nausea and vomiting, it is recommended that patients take TMZ at least 1 h before a meal or, preferably, at bedtime.

Phase I and II clinical studies have determined the maximum tolerated dose to be 1,000 mg/m^2^ divided over five days in each 28-day cycle [1,2,3,4,5,6]. TMZ is generally well tolerated at these dose levels. The most common nonhematologic adverse events are nausea, vomiting, headache, fatigue, and constipation. These events are generally mild to moderate in severity, and nausea and vomiting are readily controlled with standard antiemetics. With regard to hematologic toxicities, the incidence of grade 3/4 neutropenia and thrombocytopenia is generally lower than 10%, and fewer than 10% of patients required hospitalization, blood transfusion, or discontinuation of therapy due to myelosuppression. Myelosuppression is as a rule not cumulative and rarely results in discontinuation of treatment.

Based on the favorable toxicity profile observed in early clinical trials, subsequent studies have investigated doses of 150 mg/m^2^/day χ 5 days for patients who had previously been treated with cytotoxic chemotherapy (total dose = 750 mg/m^2^ per cycle), with a planned increase to 200 mg/m^2^/day if no major myelosuppression was evident on day 22 of the 28-day cycle. For previously untreated patients, the initial dose is typically 200 mg/m^2^/day χ 5 days (total dose = 1,000 mg/m^2^ per cycle). A continuous oral dosing schedule has also been investigated in a phase I trial [44]. Administration of 75 mg/m^2^/day for six to seven weeks was well tolerated and resulted in a twofold increase in drug exposure over four weeks compared with the five-day every 28 days schedule. This regimen produced a 33% objective response rate among 24 patients with varying tumor types, including 17 patients with gliomas. The continuous daily regimen lends itself to potential combination with fractionated radiation therapy.

**Figure 2:**
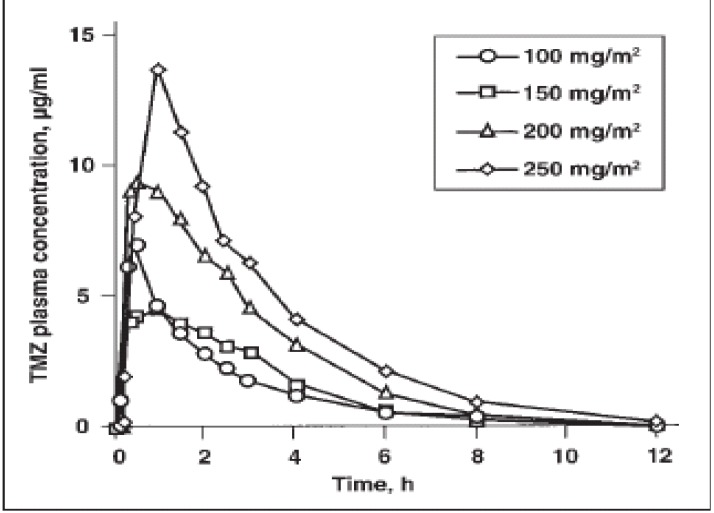
Shows the plasma concentration during time after oral administration of a single dose of temozolomide ranging from 100 to 250 mg/m^2^ [46].

The pharmacokinetics of TMZ after administration at 150 and 200 mg/m^2^ on a five-day schedule have been evaluated in humans (Table 1) [45]. The data indicate that TMZ is rapidly absorbed and eliminated. The mean time to maximal plasma concentration was less than 1 h, and the mean terminal elimination half-life was approximately 1.8 h. The maximum plasma concentration was 7.75 and 10.7 mg/ml at the 150 and 200 mg/m^2^ doses, respectively. Figure 2 [46] shows the plasma concentration curve after oral administration of a single dose of TMZ ranging from 100 to 250 mg/m^2^. Nearly all of the administered dose was eliminated within 8 h. Because of its rapid elimination and its mechanism of action, TMZ has a reduced risk of causing cumulative hematologic toxicity. The mean plasma area under the curve of TMZ after oral administration was 22.6 and 29.7 mg·h/ml after a dose of 150 and 200 mg/m2, respectively. These pharmacokinetic characteristics have been evaluated in a number of clinical studies and are consistent among studies and in different patient subgroups, including pediatric patients [5,47,48,49].

**Table 1:** Pharmacokinetics after single dose of temozolomide [45].

	Oral dose	
Parameter	150 mg/m^2 ^(n=12)	200 mg/m^2 ^(n=6)
C_max_	7.75 μg/mL	10.7 μg/mL
T_max_	0.85 h	0.89 h
AUC	22.6 μg·h/mL	29.7 μg·h/mL
T_1/2_	1.81 h	1.84 h
Clearance	3.05 mL/kg/min	2.85 mL/kg/min
Volume of distribution	0.48 L/kg	0.45 L/kg

C_max_: maximum plasma concentration; T_max_: time of maximal concentration; AUC: area under the curve; T_1/2_: terminal elimination half-life.

## Temozolomide in Malignant Gliomas

Generally, HGGs have a poor prognosis, are rapidly progressive and resistant to therapy. Due to the fact that, they have an infiltrating they cannot be completely excised and the majority will recur within 2 cm of their original location. Median survival is around one year for GBM (gliblastoma multiform), two years for AA (anaplastic astrocytoma) and five years for AO (anaplastic oligodendroglioma) [50]. The main scope of the management is to achieve a symptomatic relief and increasing survival. The first option is surgery, which is usually required in some form for histological diagnosis. This may involve a biopsy or more aggressive resection. Currently there is no good evidence from RCTs (randomized controlled trails) that either approach results in any difference in survival over best medical care, although resection is commonly attempted where feasible [51]. Radiotherapy is the treatment with the greatest evidence base for effect and this is now part of standard management, resulting in an increase in median survival from three to four months to around nine to ten months [52]. The other principle therapy is glucocorticosteroids, which have an important role in the reduction of peri-tumoural oedema and can produce a marked improvement in neurological symptoms and survival by themselves [53].

Chemotherapy has been used as part of initial therapy as either single agent or multi-agent regimes to try and maximise penetration through the blood brain barrier and tumour responsiveness. The results have generally been interfering, and there is a meaningful morbidity associated with chemotherapy [54]. Recently a meta-analysis of chemotherapy in HGG has demonstrated an improvement in survival with PCV (procarbazine, carmustine, vincristine) chemotherapy (HR 0.85 CI 0.78 to 0.92 p < 0.0001) with an overall improvement of two months in median survival to around 12 months [55]. It is not clear whether the gain in survival reflects a useful period of good QOL. In grade III tumours two recent RCTs did not demonstrate an increase in survival with PCV [56,57].

Temozolomide is a chemotherapeutic drug which is administered orally that methylates DNA in a way which prevents tumour cell proliferation. Early case series have suggested temozolomide to be a safe therapy with haematological toxicity in five to ten per cent, as well as being associated with a good median survival of around 16 months [58,59]. These survival figures compared favourably with expected prognosis in GBM, while toxicity was lower than with high dose procarbazine chemotherapy. Temozolomide is rapidly becoming standard therapy for GBM in the primary and recurrent disease settings, and has recently been licensed by the National Institute of Clinical Excellence in the UK in these disease settings. This practice has largely been based on a single high profile RCT although there is no full systematic review and meta-analysis in the field to fully assess the evidence basis for this trend in clinical practice.

### Combination of Temozolomide with Radiation Therapy

TMZ alone has only a modest effect on high-grade glioma. It is mainly the interaction between TMZ and radiation therapy which is critical in the treatment of high grade glioma, especially in glioblastoma. The improvement effectiveness of combined TMZ and radiation therapy in patients with malignant glioma in comparison with radiation therapy alone was demonstrated in several preclinical and clinical studies. Wedge *et al* [60] have studied the in vitro cytotoxicity of 8-carbamoyl-3-methylimidazo [5,1-d]-1,2,3,5-tetrazine-4(3H)-one (temozolomide) with concurrent X-irradiation in a human glioblastoma cell line (U373MG) as a potential radio-chemotherapeutic treatment for malignant glioma. The authors have concluded that the combination of temozolomide with radiation is at best additive, but could nonetheless by of considerable therapeutic benefit in glioma, particularly if administered for prolonged periods. Moreover, Hirose *et al.* [61] have studied the action of TMZ in gliomas and the role p53 might play by using U87 glioma cells that were either p53-wild-type or p53- deficient (by virtue of expression of the viral oncoprotein E6). They have shown that glioma cells respond to TMZ by undergoing G2-M arrest. p53 is not necessary for this G2-M arrest to occur but is important in the duration of G2-M arrest and in the ultimate fate of TMZ-treated cells. Therefore, the integrity of the G2-M cell cycle checkpoint may be important in the cytotoxicity of TMZ in glioma cells. Additionally, Chakravarti *et al.* [62] have investigated the mechanisms by which temozolomide enhances radiation response in glioblastoma cells. They have shown that temozolomide enhances radiation response most effectively in *O*^6^-methylguanine-DNA methyltransferase (MGMT)-negative glioblastomas by increasing the degree of radiation-induced double- strand DNA damage. In MGMT-positive glioblastomas, depletion of MGMT by the addition of *O*^6^-benzylguanine significantly enhances the antitumor effect of concurrent radiation + temozolomide. Finally, Stupp *et al.* [63] recently have demonstrated that in a longer follow-up of five years, the patients with glioblastoma that received combined treatment with TMZ and radiation therapy have significantly better overall survival when compared with those that received radiation therapy alone. A few patients in favourable prognostic categories survive longer than five years. MGMT methylation status identifies patients most likely to benefit from the addition of temozolomide.

The main objective of this review article was to estimate the effectiveness of temozolomide in therapy of HGG as part of a) primary treatment and b) in recurrent disease. We have incorporated as eligible all randomized controlled trails that were consisted of two arms: first arm, intervention, patients that had received radiation therapy comcomitant with temozolomide and second arm, comparison, patients that had received radiation therapy only or radiation therapy in association with other form of chemotherapy e.g. PCV, Gliadel. The primary outcomes were survival from time to randomization to time of death, and secondary were: time to progression, quality of life and adverse events.

### Eligible trials

As regarding the effectiveness of temozolomide in therapy of HGG as part of primary treatment the literature research had shown that Two RCTs met the inclusion criteria. The first trial, involving 85 centres in 15 countries, was run by the EORTC (European Organization for Research and Treatment of Cancer) from August 2000 to March 2002 [64]. It evaluated concomitant and adjuvant temozolomide with radiotherapy in comparison with radiotherapy alone in primary therapy for histologically confirmed GBM only. Randomisation was adequate but the trial was not blinded and did not include a placebo. A total of 573 patients with newly histologically diagnosed GBM (at biopsy or resection) were included with inclusion criteria specifying age between 18 to 70 and a WHO (World Health Organization) performance score of 2 or less. Standard radiotherapy schedules of 60 Gy for six weeks were given to both arms. The temozolomide schedules was 75 mg/m^2^ daily during radiotherapy then up to 6 adjuvant cycles of 150 to 200 mg/m^2^ for five out of 28 days for a total of six cycles. No routine chemotherapy was given to the control arm. Subsequent management was given according to need with no pre-specified protocol mentioned. The primary end point was overall survival. Secondary endpoints included progression free survival and safety. A follow up article was published describing only the QOL results from the initial trial run by the EORTC [65]. This used the EORTC QLQ-C30 and QLQ-BCM20 as combined outcome measures which were then converted to a score of 0 to 100. The difference between groups and from baseline was recorded for seven groups (overall, fatigue, social function, emotional function, future uncertainty, insomnia and communication deficit). Compliance with questionnaires was also recorded. The second trial was also published in 2005 and was set in Greece [66]. It used a dose intensification schedule of temozolomide in the adjuvant phase involving 150 mg/m^2^ of temozolomide on days 1 to 5 and 15 to 19. In the concomitant phase temozolomide was administered using a standard 75 mg/m^2^. Radiotherapy was administered to both arms in a dose of 60 Gy over 6 weeks. Randomisation was adequate but the trial was not blinded and did not include a placebo. A total of 130 patients were randomised, with inclusion criteria specifying GBM on histology, Karnofsky Performance Scale of 60 or more, and age over 18 (but with no upper age limit specified). Poor medical condition was an exclusion factor. Primary outcome measures were overall survival and time to progression. Secondary outcome measures included toxicity.

For recurrent disease we have found only one eligible trial that was a randomised, multicentre, open-label phase II study comparing temozolomide with procarbazine for first relapse of GBM [67]. A total of 225 patients were randomised between January 1995 and October 1997 and subject to an intention to treat analysis. Randomisation was deemed to be adequate but was not blinded. Temozolomide was administered on days 1 to 5 out of 28 and procarbazine administered for 28 days out of every 56. No radiotherapy was administered to either arm. Primary outcome measures were progression free survival and overall survival. Secondary outcome measures were health related QOL (measured by EORTC QLQ-C30 and QLQ-BCM20), objective responsiveness and toxicity/adverse events. Table 2 summarizes the characteristics of eligible trials for both primary and recurrent disease.

**Table 2:** Characteristics of eligible trials.

*Primary therapy*				
Author	Year published Type of trial	Number of patients	Treatment schecule	Outcomes
Athanasiou [66]	2005, RCT	130	Concomitant TMZ daily with RT for 6 weeks. Adjuvant TMZ on days 1-5 and 15-19 for less than 6 cycles.	Primary: Survival; PFS. Secondary: Safety
Stupp [64]	2005, RCT	573	Concomitant daily TMZ (75mg/m2/day) during RT (<7weeks). Adjuvant TMZ for first 5 days out of 28 for 6 or fewer cycles. RT was 60Gy focally to the tumour and a 2-3cm margin over 30 sessions and 6 weeks.	Primary: Survival. Seconday: PFS, Safety, QoL
***Recurrent disease***				
Yung [67]	2000, RCT	225	Temozolomide: 200mg/m2/day (if chemotherapy naive) or 150mg/m2/day (if prior chemotherapy) for 5 days out of a 28 day cycle. Procarbazine 150mg/m2/day (chemotherapy naive) or 125mg/m2/day (if prior chemotherapy) for 28 consecutive days in a 56 day cycle	Objective response, sixmonth PFS, median PFS, survival, adverse events

**Abbreviations**: RCT: randomized controlled trials, TMZ: temozolomide, PFS: progression free survival, RT: radiation therapy, QoL: quality of life.

## Results

For primary therapy temozolomide resulted in an increase in survival (HR 0.84, 95% CI 0.50 to 0.68, p < 0.001) compared with the control arm. Fixed effects models were used as the entry criteria for each study were broadly inclusive. As only two trials were included and both demonstrated a survival benefit individually it was apparent that there was no gross heterogeneity between the trials and a formal statistical test of heterogeneity was deemed unneccessary. There was also an increase in time to progression with temozolomide (HR 0.52, 0.42 to 0.64, p < 0.001). For QOL there was no difference between the two arms for any of the seven outcome measures (overall, fatigue, social function, emotional function, future uncertainty, insomnia and communication deficit). Due to the low level of adverse events these are presented in a descriptive manner. In the trial Stupp *et al.* [64] grade 3/4 haematologic toxicity occurred in 7% during concomitant therapy and 14% in the adjuvant phase. Events lead to treatment discontinuation in 8%. No haematologic toxicity was noted in the control arm. No statistical difference in severe infections, fatigue or thromboembolic events was reported between either arm. Athanassiou *et al.* [66] grade 3/4 haematologica toxicity was reported in the concomitant phase as leucopenia in 3.5% and thrombocytopenia in 5.2%. In the adjuvant phase the results were leucopenia in 2% and thrombocytopenia in 5%. Non-haematological side effects were rash 5%, constipation 3.5% and arthralgia 1.5%.

As regarding therapy for recurrent diseas, temozolomide was effective in increasing time to progression (HR 0.68, CI 0.51 to 0.90, p = 0.008) but not in increasing survival (HR 0.87, CI 0.65 to 1.16, p = 0.34). No appropriate data was available for calculation of QOL outcomes. Adverse events were noted in 77% of the temozolomide group and 76% of the procarbazine group. Grade 3/4 adverse events were found in 18% of the temozolomide group and 25% of the procarbazine group. Haematological adverse events were low at fewer than 7% in both groups but were not cummulatively compared; there did not appear to be a difference in the individual subgroups of haematological adverse events. Drop outs due to adverse events were: temozolomide 3% versus procarbazine 11%. The most common side effecs of all grades were: nausea (temozolomide 38% versus procarbazine 34%), vomiting (temozolomide 32% versus procarbazine 27%) and fatigue (temozolomide 27% and procarbzine 15%).

## Conclusions

Temozolomide is an effective p.o. administered anticancer agent that demonstrates a broad spectrum of activity in various solid tumors and distribution to all tissues, including the brain. It spontaneously converts to an active methylating agent with activity against a number of refractory cancers, including malignant glioma, metastatic melanoma, and other solid tumors. Temozolomide is well tolerated, with minimal myelosuppression that is noncumulative and with nonhematological toxicity that is easily managed with standard antiemetic agents. Unique characteristics of stability and solubility allow temozolomide to be absorbed readily and distributed to all tissues with approximately 100% bioavailability after p.o. administration. Thus, temozolomide does not require hepatic metabolism for activation and is capable of penetrating the blood-brain barrier. Temozolomide demonstrates dose-linear PK (pharmacokinetic), is cleared rapidly, and does not accumulate with repeat dosing. Its PK yields little intrasubject or intersubject variability, which is manifested by its predictable clinical tolerance and mild side effect profile.

The literature research indicates that temozolomide is effective as primary therapy for GBM. It prolongs survival and time to progression without a significant risk of early adverse events. It appears to be most effective in young and fit patients with GBM who have had debulking surgery. These results are based on two RCTs of 703 patients in total. Recently, the updated Phase III trial by the European Organisation for Research and Treatment of Cancer (EORTC) and National Cancer Institute of Canada Clinical Trials Group (NCIC) reported a benefit of combined therapy in all clinical prognostic subgroups, including patients aged 60-70 years. Methylation of the MGMT promoter was the strongest predictor for outcome and benefit from temozolomide chemotherapy. Temozolomide is also effective as therapy for recurrent disease, where it increases time to progression without an increase in adverse events. There are still some reservations with this data as the trials were not blinded or placebo controlled, whilst quality of life data could be further expanded upon. In a well selected subgroup of patients with GBM, temozolomide vindicate consideration for use as in either primary or recurrent disease settings, but decisions need to be made on an individual patient basis as part of a multi-disciplinary meeting discussion. Further trials are needed with improved methodology, including placebo control, blinding, and the use of clear statistical reporting of outcome measures in particular for recurrence and QoL.
